# Adalimumab for AA Amyloidosis Secondary to Severe Hidradenitis Suppurativa

**DOI:** 10.1155/crin/5476216

**Published:** 2026-03-12

**Authors:** Ghalia Khellaf, Souad Chelghoum, Houria Sahel, Rym Hadj Sahraoui, Nacima Djennane, Ali Benziane

**Affiliations:** ^1^ Nephrology Department, CHU Bab El-Oued, Hôpital Mohamed Lamine Debaghine, Algiers, Algeria; ^2^ Faculty of Medicine, Université D’Alger 1, Algiers, Algeria; ^3^ Nephrology Department CHU Hussein Dey, Hopital Nafissa Hamoud Ex Parnet, Algiers, Algeria; ^4^ Dermatology Department, CHU Bab El-Oued, Hôpital Mohamed Lamine Debaghine, Algiers, Algeria; ^5^ Pathology Department, Salim Zemirli Hospital, Algiers, Algeria; ^6^ Pathology Department, CHU Bab El-Oued, Hôpital Mohamed Lamine Debaghine, Algiers, Algeria

**Keywords:** AA amyloidosis, adalimumab, hidradenitis suppurativa, Hurley staging, nephrotic syndrome, screening for proteinuria, TNF-α inhibitor

## Abstract

Hidradenitis suppurativa (HS) (Verneuil’s disease) is a chronic inflammatory cutaneous disorder with systemic inflammatory potential. A rare and severe sequela of this sustained inflammation is reactive (AA) amyloidosis, typically presenting with nephrotic‐range proteinuria and progressive renal failure. Here, we report a 50‐year‐old male with severe (Hurley Stage III) HS who developed nephrotic syndrome secondary to biopsy‐proven renal AA amyloidosis. Initiation of the TNF‐α inhibitor adalimumab induced a dramatic concurrent improvement in both cutaneous and renal disease activities. Proteinuria declined from a baseline of 9/24 to 2 g/24 h at 4 months and subsequently to 0.9 g/24 h at 10 months, signifying a near‐complete nephrotic remission.

**Discussion:**

This case underscores the dual therapeutic mechanism of TNF‐α blockade in this context: reducing systemic inflammation and preventing amyloid deposition, with improvement in renal prognosis. There is literature corroborating the usefulness of TNF‐α inhibitors in early‐stage AA amyloidosis. The application of adalimumab early in severe forms of HS may avert AA amyloidosis–related renal involvement or renal function decline. We suggest implementing systematic screening for significant proteinuria in patients with advanced HS (Hurley II/III) to facilitate timely diagnosis and therapeutic intervention to preserve renal function.

## 1. Introduction

The etiological diagnosis of AA renal amyloidosis remains challenging due to its association with diverse chronic inflammatory diseases. Recent data, including from our department (2023) [1], indicate a decline in idiopathic cases, reflecting improved diagnostic vigilance. Hidradenitis suppurativa (HS), a prevalent (≈ 1%) yet frequently overlooked chronic inflammatory dermatosis [2], exemplifies an underrecognized etiology. Diagnostic delays in HS are common, leading to protracted, uncontrolled inflammation and elevating the risk of severe systemic complications such as secondary amyloidosis. For years, management of severe HS was limited to symptomatic therapies (e.g., prolonged antibiotics and corticosteroids), lacking disease‐modifying options. This paradigm shifted following the PIONEER trials [3], which established adalimumab’s efficacy and led to its regulatory approval for moderate‐to‐severe HS. We here present one of the first nationally documented cases of biopsy‐proven AA renal amyloidosis secondary to severe HS, successfully treated with adalimumab, illustrating the critical intersection of timely dermatological intervention and nephrological outcome.

## 2. Case Report

**FIGURE 1:**
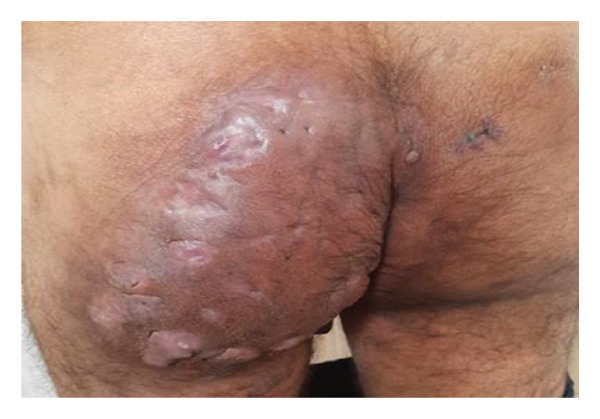
Pretreatment: Hypertrophic scarring and multifistulated nodules on the left buttock.

**FIGURE 2:**
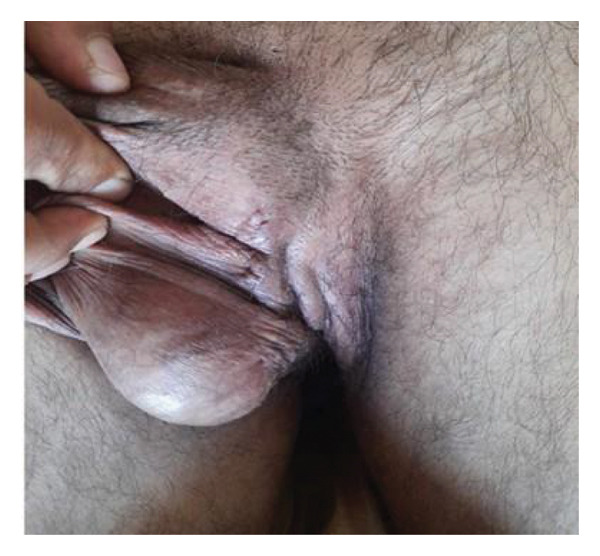
Pretreatment: Inflammatory nodules and lesions in the inguinal fold.

A 50‐year‐old male presented with a 15‐year history of severe HS, characterized by recurrent painful nodules and draining fistulas affecting the left buttock, right buttock, and inguinal folds. Physical examination revealed a multifistulated nodular plaque on the left and hypertrophic scarring on the right buttock (e.g., Figures 1 and 2). Based on these findings, a diagnosis of Hurley Stage III HS was established. The patient was hemodynamically stable at presentation.

Dipstick urinalysis showed 4+ proteinuria with a pH of 6 and no other remarkable findings. Subsequent laboratory investigations revealed markedly elevated inflammatory markers, including a C‐reactive protein (CRP) of 150 mg/L, hemoglobin of 10 g/dL, hypoalbuminemia (serum albumin of 8.9 g/L), and leukocytosis of 14,000/mm^3^ (with 10,000/mm^3^ neutrophils), and preserved renal function at baseline (serum creatinine of 1.5 mg/dL and estimated glomerular filtration rate [eGFR] of 100 mL/min/1.73 m^2^). A 24‐hour urine collection confirmed nephrotic‐range proteinuria of 9 g/24 h.

**FIGURE 3:**
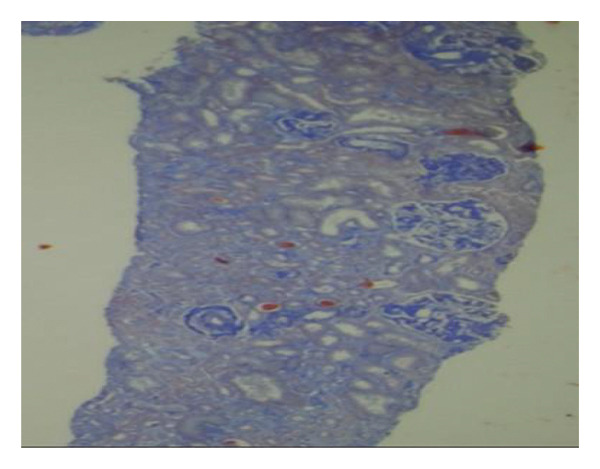
Renal biopsy: Congo red stain showing glomerular amyloid deposits with characteristic apple‐green birefringence under polarized light.

A histological study of a percutaneous renal biopsy was performed for evaluation. Light microscopy after Masson’s trichrome staining revealed a cortical core containing 14 glomeruli, including 3 globally sclerosed. All glomeruli are pathological, showing acellular deposits with Congo red stain and green birefringence under polarized light, leading to mesangial expansion without mesangial hypercellularity. Capillary lumina are open. Tubules and interstitium: presence of fibrosis estimated between 10% and 15% with associated tubular atrophy. Presence of clusters of foamy interstitial histiocytes and hyaline casts, sometimes with a pseudocystic appearance. Vessels: no large‐caliber vessels; presence of four medium‐caliber arteries containing the same deposits as described above. Interlobular arterioles without abnormalities. Immunohistochemistry stains showed that the amyloid deposits were AA‐type (e.g., Figure 3).

**FIGURE 4:**
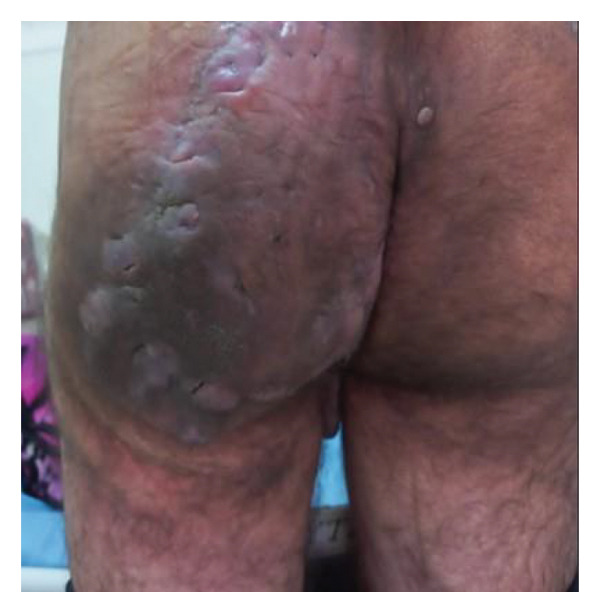
Posttreatment: Reduction in fistulas and scarring after 3 months of adalimumab therapy.

**FIGURE 5:**
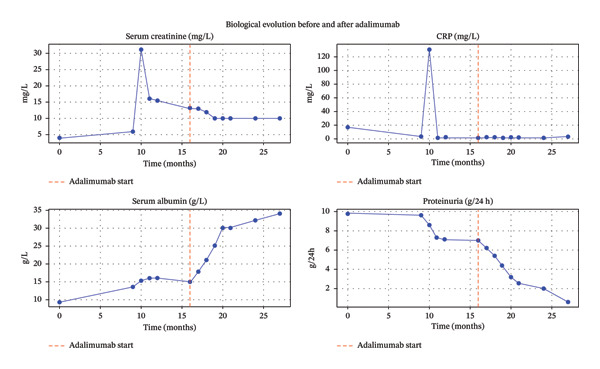
Clinical and biological evolution of the patient before and after adalimumab treatment, showing the decline in proteinuria and stability of renal function.

Initially, the patient was started on TNF‐α inhibitor adalimumab. The patient received a loading dose of 80 mg 2 weeks after starting therapy, followed by a maintenance regimen. Concurrent medications included angiotensin II receptor blockers and antibiotics. Within three months of therapy, a marked improvement in skin lesions was observed (Figure 4), accompanied by a reduction in proteinuria to 5 g/24 h. After 10 months of adalimumab therapy and a total follow‐up of 26 months, proteinuria further decreased to 0.9 g/24 h, with preserved renal function as serum creatinine of 1.0 mg/dL and an eGFR of 84 mL/min/1.73 m^2^ (Figure 5).

## 3. Discussion

### 3.1. Etiology and Pathophysiology of HS and Renal AA Amyloidosis

HS is a chronic inflammatory disease with a multifactorial etiology, involving genetic predisposition and lifestyle factors such as obesity and smoking [4]. The disease originates in the pilosebaceous unit of intertriginous areas, where follicular occlusion and hyperkeratinization occur. Subsequent follicular rupture releases keratin debris and bacterial components, activating innate immune pathways including the NLRP3 inflammasome and leading to the release of proinflammatory cytokines such as IL‐1β and TNF‐α [2]. This initial response is followed by an adaptive immune reaction involving IL‐17 and IFN‐γ producing T cells, alongside B cells within tertiary lymphoid structures [5]. This sustained inflammatory state, mediated by a dysregulated cytokine cascade (TNF‐α, IL‐1β, and IL‐6) [6], drives hepatic overproduction of serum amyloid A (SAA) [6, 7]. Chronically elevated SAA levels (> 100 mg/L) facilitate its proteolytic cleavage into amyloidogenic fragments, culminating in systemic tissue deposition and AA amyloidosis [8]. These SAA remnants latch onto substances (glycosaminoglycans or GAGs) within the kidney’s filtering structures. Once settled, they undergo a transformation, morphing into rigid, sheet‐like formations: amyloid fibrils [9]. These amyloid fibrils amass in specific areas of the kidney, disrupting the cells responsible for filtration and causing protein to escape into the urine (9 g/24 h in our patient). The kidney biopsy confirmed these amyloid clusters within the filtering units, explaining the significant protein loss in the urine, even with relatively good kidney function (eGFR of 95 mL/min). HS‐driven inflammation (TNF‐α, IL‐1β, and IL‐17) elevates SAA (> 100 mg/L) [10]. SAA is processed by macrophages into amyloidogenic fragments [9], forming amyloid fibrils [11]. These deposits disrupt filtration, causing proteinuria, an early warning sign [11], and trigger TGF‐β‐mediated fibrosis [12]. AA amyloidosis progresses faster to renal failure (2‐3 years) than AL/ATTR variants [13].

### 3.2. Adalimumab in HS and Associated Renal Complications: An Integrated Pathophysiological Approach

The management of moderate‐to‐severe HS was revolutionized by the introduction of adalimumab, a monoclonal anti‐TNF‐α antibody that neutralizes this pivotal cytokine, marking a paradigm shift in treatment [2, 14]. Therapeutically, adalimumab acts by specifically inhibiting TNF‐α. This targeted blockade significantly reduces systemic inflammation and the hepatic synthesis of acute‐phase reactants, most notably SAA [14]. By mitigating the chronic SAA elevation that drives AA amyloid deposition, TNF‐α blockade is postulated to interfere directly with amyloid fibrillogenesis.

The renal improvement observed in our case aligns with growing clinical evidence supporting this pathophysiological rationale. Multiple reports describe the stabilization or regression of AA amyloidosis with anti‐TNF‐α therapy, particularly when initiated early. For instance, Helvacı et al. documented several patients with HS‐associated renal AA amyloidosis who achieved clinical stabilization or improvement following biologic treatment [15]. This is corroborated by individual case reports, including one describing complete clinical remission of nephrotic syndrome in an HS patient after adalimumab [16] and another demonstrating marked renal stabilization with infliximab [17].

### 3.3. Current Therapeutic Hierarchy and Future Perspectives

Long‐term antibiotics with anti‐inflammatory properties (e.g., tetracyclines and amoxicillin–clavulanic acid) are the initial cornerstone for managing inflammation. For moderate‐to‐severe cases where antibiotics fail, adalimumab, an anti‐TNF‐α biologic, is the first‐line and best‐supported biologic therapy. For cases of anti‐TNF failure or intolerance, IL‐17 inhibitors (e.g., secukinumab and bimekizumab) and JAK inhibitors represent primary alternatives [18]. Treatment selection should be personalized, incorporating the patient’s clinical profile, including the presence of sinus tunnels, pain characteristics, comorbidities, and history of infection [19].

### 3.4. Efficacy and Limitations

Adalimumab is substantially efficacious for HS treatment. Approximately 59% of patients have significant improvement in dermatology after 12 weeks of treatment, with some pediatric studies suggesting improvement rates upwards of 84% over longer treatment periods. For amyloidosis, approximately 68% of patients have a reduction in proteinuria (≥ 50%). Of note, there is a subset of patients (30%–40%) that do not respond to adalimumab and who may require alternative therapy (i.e., IL‐17 inhibitors) [14, 20]. Cost: Adalimumab is an expensive medication, which can be a barrier to access for many patients. However, the increasing availability of biosimilars is helping to lower costs and improve accessibility to the treatment.

## 4. Limitations

While this case demonstrates promising outcomes with adalimumab therapy, there are several limitations that warrant consideration. Firstly, the single‐case design inherently limits the generalizability of the findings. Secondly, although the significant decrease in proteinuria (from 9/24 to 0.9 g/24 h) strongly indicates amyloid regression, the lack of a follow‐up renal biopsy prevents definitive histological confirmation of amyloid clearance. Thirdly, although our 2‐year follow‐up shows sustained renal improvement, long‐term monitoring would be valuable in assessing the durability of the response and the potential for late recurrence. Finally, due to resource constraints, SAA levels, the gold‐standard biomarker for AA amyloidosis activity, were unavailable, necessitating the use of CRP as a surrogate inflammatory marker.

## 5. Conclusion

This case report and review of relevant literature provide evidence that early and aggressive adalimumab therapy in patients with severe HS can lead to significant reduction of renal complications, namely, nephrotic syndrome due to AA amyloidosis. The decreased urinary protein excretion noted in the case suggests the high impact of early intervention. Therefore, annual urine screening should be implemented in all high‐risk HS patients. Early TNF‐α inhibition may reverse proteinuria in amyloidosis, underscoring the value of nephrologist–dermatologist collaboration for high‐risk HS patients. Future studies should explore other biomarkers for early detection and other treatment options for those patients with treatment resistance.

## Author Contributions

G.K.: diagnosis of hidradenitis suppurativa, project administration, writing–original draft, and writing–review and editing. S.C.: writing and review. H.S.: clinical management of the patient and resources. R.H.S.: histopathological analysis and interpretation of the renal biopsy. N.D.: supervision of the histopathological analysis and resources. A.B.: performance of the renal biopsy, supervision, and validation.

## Funding

No funding was received for this manuscript.

## Ethics Statement

Information provided in this paper is in compliance with ethical guidelines and in accordance with the World Medical Association Declaration of Helsinki. Ethical approval is not required for this study in accordance with local or national guidelines. Written informed consent was obtained from the patient and his brother for publication of the details of their medical case and any accompanying images.

## Conflicts of Interest

The authors declare no conflicts of interest.

## Data Availability

Data sharing is not applicable to this article as no new data were created or analyzed in this study.
